# Genome-wide mapping of DCP2-dependent 5′ cap footprints in *Arabidopsis thaliana*

**DOI:** 10.1093/nargab/lqag088

**Published:** 2026-08-10

**Authors:** Neha Shukla, Michael A Schon, Vivek K Raxwal, Michael D Nodine, Karel Riha

**Affiliations:** Central European Institute of Technology, Masaryk University, Brno 62500, Czech Republic; Laboratory of Systems and Synthetic Biology, Wageningen University, Wageningen 6708 PB, the Netherlands; Central European Institute of Technology, Masaryk University, Brno 62500, Czech Republic; Laboratory of Molecular Biology, Wageningen University, Wageningen 6708 PB, the Netherlands; Central European Institute of Technology, Masaryk University, Brno 62500, Czech Republic

## Abstract

Messenger RNA (mRNA) decapping mediated by DCP2 is a key mechanism controlling RNA stability and gene expression in eukaryotes, including plants. Despite its central role in regulating plant development and stress responses, the repertoire of mRNA 5′ caps targeted by DCP2 remains undefined. Here, we combined *in vitro* decapping with 5′-end enriched and full-length transcriptome sequencing of DCP2-deficient mutants to comprehensively characterize the mRNA capping landscape in *Arabidopsis thaliana*. We mapped over 13 000 high-confidence capped transcripts at nucleotide resolution, revealing distinct 5′ cap signatures in wild-type and *dcp2* seedlings. Most caps localized near annotated transcription start sites, validating the accuracy of our approach. Loss of DCP2 led to substantial expansion of detectable capped 5′-ends, including 275 caps from previously unannotated loci, and an increased prevalence of multi-capped genes, highlighting the role of DCP2-mediated decapping in removing unwanted transcripts. Integration with degradome resources revealed extensive convergence between DCP2-sensitive capped RNAs and substrates of co-translational, cytosolic XRN4-dependent decay, as well as nonsense-mediated decay pathways. Our results suggest that DCP2 shapes the steady-state 5′ cap landscape *in vivo* by limiting the accumulation of unstable and cryptic capped transcripts. Also, this study provides a valuable resource for transcript annotation and isoform-aware analysis of RNA turnover.

## Graphical Abstract

**Graphical Abstract:**
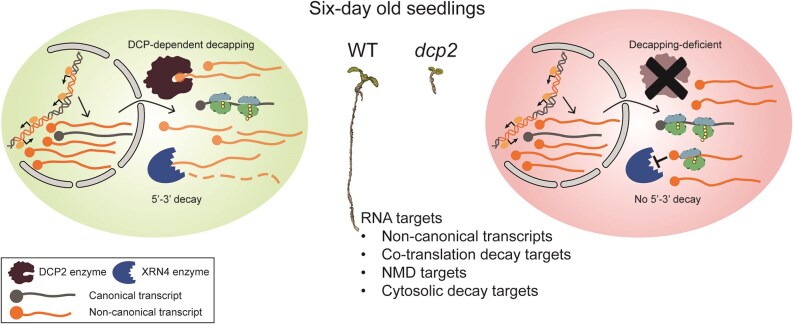

## Introduction

Gene expression is largely determined by the steady-state levels of mature messenger RNA (mRNA), which arise from balance between RNA synthesis and decay [1, 2]. Cytosolic mRNA decay is not a random process but proceeds through specific, well-defined pathways [3–5]. In the predominant 5′–3′ decay, deadenylation is followed by removal of the protective 5′ cap (m^7^GpppN) through decapping and subsequent exonucleolytic degradation by ribonuclease XRN1 or its plant homologue XRN4 [4, 6–8]. Decapping is responsible for the turnover of bulk cytoplasmic mRNA [9–11], as well as transcripts targeted by specialized surveillance mechanisms, including nonsense-mediated decay (NMD) [12–14], microRNA-mediated decay [15, 16], degradation of AU-rich elements-containing mRNAs [17], and transcripts with non-optimal codons [3, 18]. Thus, decapping represents a key step in mRNA turnover, making it central to the post-transcriptional regulation of gene expression.

The cleavage of 5′ caps is mediated by the DCP1/2 decapping complex [19]. The primary catalytic subunit of this complex is DCP2, whose activity is enhanced by DCP1 [20–23]. DCP2 is a member of the Nudix (nucleoside diphosphates linked to moiety X) hydrolase family [24–26]. Among numerous Nudix hydrolases, DCP2 exhibits selective activity toward m^7^G-capped mRNAs by forming a cytoplasmic multi-protein complex with other decay factors [27–31].

Decapping has been very well defined across all eukaryotic organisms. Decapping-deficient mutants display pleiotropic developmental and stress-responsive phenotypes, underscoring the essential role of decapping in normal physiological processes. The compromised decapping in *Caenorhabditis elegans* and *Drosophila melanogaster* results in impaired growth and development, shortened lifespan, and increased stress sensitivity [32–34]. In mice, defective decapping results in slow-growth phenotype and enhanced innate immune responses, while the postnatal depletion of DCP2 leads to male infertility [17, 35]. In plants, the core decapping complex consists of DCP1, DCP2, and scaffold Varicose (VCS), and *Arabidopsis* mutants lacking any of these genes exhibit seedling lethality [36, 37]. In addition to its role in development, decapping is important for the precise degradation of specific transcripts to ensure proper regulation of various cellular processes, especially during stress responses [6, 17, 38–46].

Analysis of RNA decay rates in *Arabidopsis vcs-7* null mutants suggests that decapping contributes to the decay of up to 68% of the transcriptome [10]. Despite extensive genetic and biochemical characterization of the decapping machinery, the transcriptome-wide landscape of DCP2-dependent cap states remains poorly defined. Previous studies have primarily inferred decapping through the accumulation of downstream decay intermediates or through transcript stabilization phenotypes observed in decapping-deficient backgrounds, rather than by directly mapping DCP2-dependent cap states genome-wide [10, 36, 47–49]. Likewise, genome-wide mapping of transcription start sites (TSSs) has established the promoter architecture with TSS tag clusters, but do not directly provide information on decapping-dependent transcript abundance [50]. Consequently, the *in vivo* substrates of DCP2 and the precise positions of canonical 5′ caps, have not been comprehensively mapped.

Here, we use low-input mRNA 5′-end enriched sequencing [51], in combination with *in vitro* decapping, to accurately delineate 5′ cap footprints of mRNAs in *Arabidopsis* seedlings. Comparison of wild type and *dcp2* mutants revealed transcripts subjected to decapping-mediated turnover and distinguish canonical TSSs from caps associated with post-transcriptional regulation. Our data provide an important resource for understanding the role of mRNA decay in transcriptome homeostasis.

## Materials and methods

### Plant materials and growth conditions

*Arabidopsis thaliana* Columbia-0 ecotype was used as wild type. The T-DNA insertion mutants, *dcp2-1* (SALK000519), *dcp1-1* (GK-844B03.02), *vcs-6* (SAIL_831_D08), *vcs-7* (SALK032031), and *eds1-2* [52] used in this study were obtained from NASC. Seeds were sterilized and plated on agar plates containing half-strength Murashige and Skoog medium, adjusted to pH 5.7, supplemented with 1% (w/v) sucrose. Plates were cultivated in growth chambers under 16/8-h light/dark cycle at 22°C. All the decapping-deficient single or double mutants used in the study were polymerase chain reaction (PCR) genotyped to confirm the presence of T-DNA insertions (Supplementary Table S1).

### *In vitro* decapping assay, library construction, and sequencing

For RNA isolation, 6-day-old seedlings of wild type and *dcp2-1* homozygous mutants were harvested. Seedlings from each plate were pooled and considered one biological replicate. For each genotype, total RNA was isolated from two independent biological replicates using RNA Blue (TOP-Bio, Catalog No. R013). Following RNA extraction, samples were treated with TURBO DNase (Thermo Fisher Scientific, Catalog No. AM2238) to remove genomic DNA contamination. The RNA was then purified using magnetic beads (Beckman Coulter, Catalog No. A63987), following the manufacturer’s instructions. Total RNA from each sample was split into two parts, one treated with DCP2 enzyme and the other with no treatment (NT).

For *in vitro* decapping assay, 5 μg total RNA samples were treated with the mRNA Decapping Enzyme (NEB, Catalog No. M0608S) at 37 degrees Celsius for 30 min. RNA was purified post-treatment using magnetic beads (Beckman Coulter, Catalog No. A63987) to ensure clean separation from the enzyme and other reaction components. Then, we used a modified low-input Parallel Analysis of RNA Ends (nanoPARE) protocol as described by Schon *et al*., 2018 [51] for library preparation. For complementary DNA (cDNA) synthesis, 1 μg treated RNA or 1 μg non-treated RNA was reverse-transcribed with anchored oligo-dT primer and template-switching oligonucleotide (TSO) oligo using SuperScript IV reverse transcriptase (Thermo Fisher Scientific, Catalog No. 18090050) using recommended cycle conditions. cDNA synthesis was followed by pre-amplification and was purified using SPRISelect magnetic beads (Beckman Coulter, Catalog No. B23318). The integrity and quality of pre-amplified cDNA were assessed by a fragment analyzer (CF Genomics, CEITEC). Following pre-amplification, 1 ng pre-amplified cDNA was used to prepare both 5′-end enriched libraries and full-length whole-transcriptome libraries. Briefly, cDNA was tagmented, and library amplification was performed using NextraXT kit (Illumina, Catalog No. FC-131-1024) following manufacturer’s protocol.

The final libraries were checked on a fragment analyzer and subsequently sequenced. Transcriptome libraries were sequenced 75-bp single-end using standard Illumina sequencing primers, and 5′-end enriched libraries were sequenced 150-bp single-end using a mix of custom sequencing primers on the Illumina NextSeq platform at CEITEC genomics core facility.

### Data processing and analysis of transcriptome reads

The low-quality reads and adapters were first trimmed from the total reads using cutadapt v4.1 [53]. Then, the RNA-Seq data were aligned to the reference AtRTD3 transcript dataset [54] and simultaneously quantified using Kallisto software v0.50.1 [55]. The downstream data normalization and differential expression analysis were done using the 3D RNA-Seq tool [56, 57].

In brief, the read counts and transcripts per million reads (TPM) were generated and normalized to transcript length over the samples and the library size using tximport R package (version 1.10.0) and lengthScaledTPM method. The low-expressed genes were filtered based on data mean-variance trend. A gene was expressed if it has at least one expressed transcript with 1 count per million mapped reads (CPM) in two or more samples. The trimmed mean of M-values method was used to normalize the gene read counts to CPM. The voom-pipeline of limma R package was used for pair-wise comparisons of gene expression between the samples. Statistical analyses were performed using Student’s *t*-test or the Wilcoxon rank-sum test, as indicated in the figure panel, to evaluate differences in expression between the samples. Corresponding *P*-values are reported in the figures. A gene was significantly differentially expressed in a sample if it had adjusted *P*-value < .05 (Benjamini–Hochberg-corrected) and absolute log_2_FC ≥ 1. The previously published RNAseq dataset for wild type and *vcs-7* (GSE86359) [10] was reanalyzed in a similar way for comparisons with present study.

### Data processing and analysis of nanoPARE reads

Sequencing data from 6-day-old seedlings generated in this study have been deposited in the Gene Expression Omnibus under accession number GSE315704. For each sample, 5′ caps were identified using the nanoPARE analysis pipeline [51]. Briefly, EndMap module removes the adapter sequences and filters low-complexity reads using cutadapt (v4.1). Then it aligns the reads to TAIR10 reference using STAR aligner (v2.7.1) with default parameters for each sample. After alignment, it applies sequence-bias correction [58] (adapted for RNA), computes per-base coverage of uniquely mapped reads, and assigns multi-mapping reads with a “rich-get-richer” scheme. The module outputs strand-specific coverage of 5′ end counts across the genome, and for 5′-end enriched libraries, it records 5′ soft-clipped bases as upstream untemplated nucleotides. Also, the known artifacts (TSO mispriming and internal oligo-dT) were masked. Furthermore, the EndGraph module evaluates each sample pair (5′-end enriched with corresponding transcriptome) to predict 5′ features per replicate with a peak at the local maxima via subtractive kernel density estimation. Briefly, to reduce expression-dependent background, the 5′ ends were normalized with corresponding transcriptome (single scale per sample). This signal was smoothed by kernel density estimation, and genome-wide discrete 5′ features (cut-off = RPM ≥ 0.5) were identified. Next, EndClass module was employed where replicates of the same sample type were merged to re-identify the peaks, and only reproducible features were retained. Features were assigned to nearby transcripts (including ≤ 500 nt upstream of 5′-terminal exons) and labeled by exon context. Later, they were classified as capped or non-capped based on the proportion of upstream untemplated guanosines (uuG) within each feature. In the present study, features with ≥5% uuG are considered capped.

### General data processing and analysis

Genomic overlaps with previously published data were performed using gene IDs, wherever available, to identify 5′ cap features of co-translational and cytosolic decay targets, NMD targets, and immune receptor transcripts. In case of unannotated loci, overlaps were performed using genomic coordinates in R (v4.4.2) with bioconductor package Genomic Ranges (v1.58.0) [59]. Shared 5′ features between samples were quantified using BED Tools intersect (v2.27.1) in a strand-specific manner [60, 61]. All the graphs and plots were generated in R (v4.4.2), and the statistical significance of feature overlaps was assessed using Fisher’s exact test. The 5′ feature overlaps between wild-type and *dcp2* samples were visualized using online interactive Venn diagram tool [62]. The 5′ cap enrichment index was calculated as a change in cap signal estimate. For each gene, we calculated the log_2_-fold change (*dcp2* versus wild type) independently using 5′-end RPM-based counts and full-length TPM-based expression. Then, the 5′ cap enrichment index was defined as the difference between these two values, indicating whether the 5′-end signal increases more than overall gene abundance.


\begin{eqnarray*}
\mathrm{Enrichment}\,\,\mathrm{index} = \mathrm{ log2}\left[ {\frac{{\mathrm{ dcp2}\,\,\left( {\mathrm{ 5p}} \right)}}{{\mathrm{ WT}\,\,\left( {\mathrm{ 5p}} \right)}}} \right]\frac{{}}{{}}\mathrm{ log2}\left[ {\frac{{\mathrm{ dcp2}\,\,\left( {\mathrm{ FL}} \right)}}{{\mathrm{ WT}\,\,\left( {\mathrm{ FL}} \right)}}} \right].
\end{eqnarray*}

Where 5p is the 5′-end expression, FL is the full-length transcript expression and WT is wild type sample.

The functional annotation of genes, their characteristics, and Gene Ontology (GO) enrichment analyses were performed online using the ShinyGO v0.81 tool [63]. False discovery rate (FDR)-adjusted *P*-values were calculated based on the hypergeometric distribution, and GO terms were considered significant if the FDR was <0.05. For clarity and better visualization, the coverage plots were edited after export from Integrative Genomics Viewer (IGV) browser [64].

## Results

### Transcriptome analysis in DCP2-deficient plants

**Figure 1.**
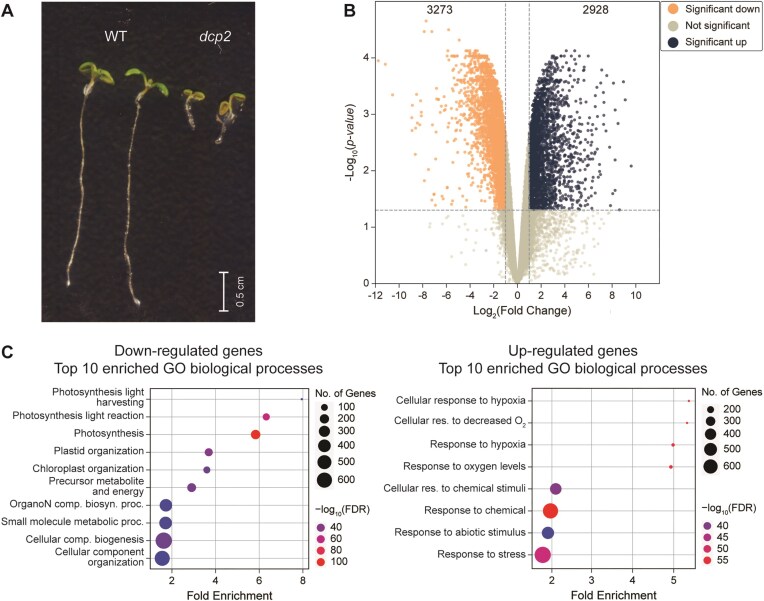
Loss of DCP2 remodels the seedling transcriptome. (**A**) Phenotypes of 6-day-old seedlings of wild type and *dcp2* null mutant. (**B**) Volcano plot of differentially expressed genes (log_2_FC) identified in *dcp2* compared with wild type. The significantly altered genes are color coded; down-regulated genes are yellow and up-regulated genes are blue. (**C**) GO enrichment analysis with dot plots showing top 10 enriched GO biological processes among down- and up-regulated genes in *dcp2* compared with wild type (adjusted *P*-value < .05). The most to least significantly enriched categories are colored as red to purple, and the size of circle correspond to the number of genes in the category. FC, fold change; GO, gene ontology; WT, wild type.

To assess the impact of decapping on the *Arabidopsis* transcriptome, we analyzed *dcp2* null mutants. Growth of *dcp2* mutants is arrested ~6 days post-germination at the cotyledon stage, leading to seedling death due to necrosis within 12–14 days post-germination [36]. Seeds from plants heterozygous for *dcp2-1* germinated on agar plates produced a fraction of growth-arrested seedlings that were validated by PCR to be homozygous mutants. Given the lethal nature of *dcp2* mutants, we analyzed the transcriptome of 6-day old seedlings, which despite growth irregularities, still exhibit green, pre-necrotic leaves (Fig. 1A). We used the SmartSeq2 method to construct the full-length poly(A) transcriptome libraries for wild type and *dcp2* mutants, each with two biological replicates.

Transcriptome analysis revealed that compared with wild type, DCP2 deficiency significantly altered the expression of 6201 genes (log_2_ fold change ≥ 1 or ≤ −1; adjusted *P*-value < .05), with 52.8% genes (3273/6201) showing decreased expression (Fig. 1B). The functional annotation using GO enrichment analysis indicated that biological processes related to stress responses, including hypoxia and abiotic stimulus, were notably enriched among up-regulated genes (Fig. 1C). Conversely, processes such as carbon fixation, photosynthesis, primary amino acid and metabolite biosynthesis, organelle, and cellular organization were significantly down-regulated in *dcp2* mutants (Fig. 1C). These signatures in gene expression are consistent with pre-senescence and growth arrest phenotype observed in the seedlings.

Similarly to *dcp2* plants, *Arabidopsis* null mutants in other components of the decapping complex also exhibit post-embryonic lethality (Supplementary Fig. S1A) [36]. To assess whether deficiency in different decapping factors elicit similar response, we re-analyzed the previously published transcriptome of *vcs-7* [10] and compared it with that of *dcp2*. As expected, a moderate correlation (Pearson’s *r*= 0.62, *n* = 17 317) was observed between the *dcp2* and *vcs-7* transcriptomes (Supplementary Fig. S1B). The GO enrichment analysis confirmed common activation of stress-responsive genes, including those involved in cellular response to hypoxia, and the suppression of genes required for generation of precursor metabolites and energy in both mutants (Supplementary Fig. S1C). These results suggest that as a fundamental process, decapping affects multiple cellular pathways.

### Mapping canonical 5′ mRNA caps

Next, we sought to complement the transcriptome analysis with a genome-wide mapping of the 5′ cap positions. To identify capped 5′ ends of mRNAs, we combined a low-input Parallel Analysis of RNA Ends (nanoPARE) sequencing [51] with *in vitro* decapping treatment using DCP2 enzyme for both wild type and *dcp2* mutant (Supplementary Fig. S2). Prior to cDNA library preparation, total RNA from each sample was split into two parts, one treated with DCP2 enzyme (T) and the other with NT. Subsequently, two cDNA libraries were created from each DCP2-treated and untreated RNA, one for 5′-end enrichment (nanoPARE) and the other for full-length transcriptome (SmartSeq2). We used SmartSeq method for library preparation because it utilizes template switching, which enriches for intact capped RNAs and enables more accurate mapping of cap-proximal positions [65]. Two biological replicas of wild-type and *dcp2* seedlings yielded a total 16 libraries, comprising ~400 million raw reads (Supplementary Table S2).

We employed the nanoPARE bioinformatics pipeline to identify 5′ ends bearing caps at single-nucleotide level resolution [51]. The pipeline identifies 5′ caps by the presence of an uuG at the junction between the genomic sequence and the TSO. Briefly, after adapter trimming and per-strand genome alignment using STAR [66], uniquely mapped reads were used to identify high-confidence 5′ ends as described in the “Materials and methods” section. We defined 5′ features representing local clusters of 5′ ends and classified a feature as capped when reads containing uuG exceeded 5% of all 5′ reads within that feature. Features falling below this threshold were considered as non-capped. This approach enabled delineation of genomic 5′ cap sites while effectively managing artifacts and biases.

**Figure 2.**
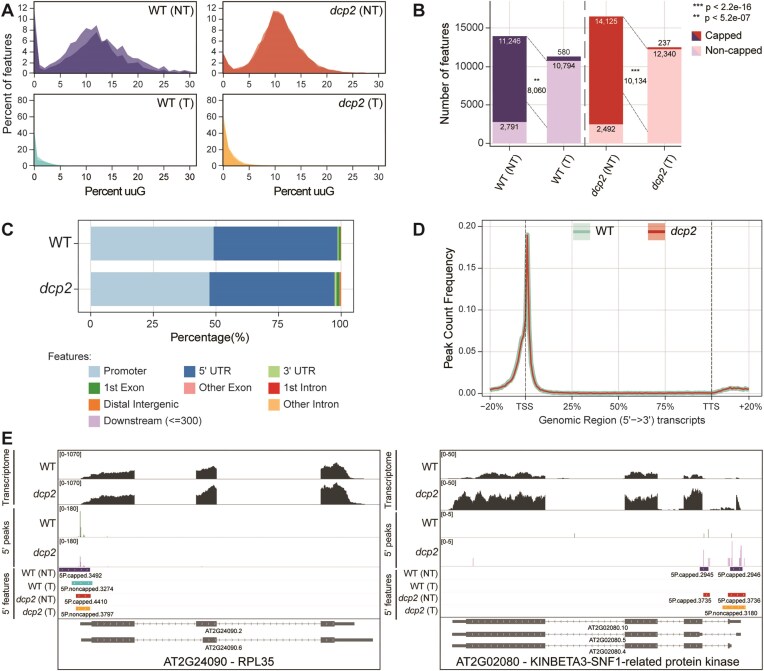
Identification of canonical 5′ features in Arabidopsis seedlings. (**A**) The distribution of 5′ ends represented as the proportion of 5′ features in nanoPARE reads (*y*-axis) with percentage of uuG (*x*-axis) from either NT or DCP2-treated fractions in both wild type and *dcp2*. (**B**) The number of 5′ features (*y*-axis) classified as either capped or non-capped, identified from wild type and *dcp2* (*x*-axis) from either NT or DCP2-treated fractions. The number of overlapping ends between the NT capped and treated non-capped in each group is indicated between the respective sample bars. The asterisk mark indicates the significance of overlap between the groups, determined by the Fisher’s exact test. (**C**) The distribution of identified 5′ caps over the genomic elements as annotated in AtRTD3 transcriptome. (**D**) The positional distribution of 5′ caps overlapping gene-body of protein-coding transcripts. (**E**) IGV browser track showing the coverage (*y*-axis) of transcriptome and 5′ peaks with example of a ribosomal protein-encoding RPL35 gene (At2g24090) and SNF1-related protein kinase (At2g02080) identified in wild type and *dcp2*. The 5′-ends as identified in each sample and the gene structure of encoded transcripts [UTRs (narrow rectangle), exons (broad rectangle), and introns (lines connecting the exons)] from IGV are displayed. IGV, Integrated genome viewer; NT, No treatment; T, DCP2-treatment; TSS, transcription start site, TTS, transcription termination site; WT, wild type.

This analysis identified a total of 19 266 5′ features from the unified NT samples of wild type and *dcp2*. Among these, 15 797 (81.9%) were classified as capped and 3 469 (18.1%) were classified as non-capped features (Supplementary Table S3). In DCP2-treated samples, the proportion of capped 5′ features decreased from 82% to <5% of all 5′ features (Fig. 2A and Supplementary Tables S4–S7). We checked the effect of *in vitro* DCP2 treatment on overall transcript abundance, and a significant correlation was observed in gene expression before and after treatment in both wild type (Pearson’s *r* = 0.97) and *dcp2* (Pearson’s *r*= 0.96) (Supplementary Fig. S3A). Also, no significantly differentially expressed genes were identified among DCP2-treated versus NT samples in both wild type and *dcp2* (Supplementary Fig. S3B), indicating that overall transcript abundance was only slightly reduced. These results indicate that *in vitro* DCP2 treatment efficiently and non-selectively removes 5′ caps without substantially affecting total RNA abundance. Further examination of the distribution of 5′ features across different samples revealed 11 246 capped sites in wild type and 14 125 in *dcp2*, whereas DCP2 treatment reduced the number of cap sites to 580 and 237, respectively (Fig. 2B and Supplementary Tables S6 and S7).

In plants, mRNA turnover is primarily influenced by decapping followed by 5′–3′ exonucleolytic degradation mediated by the cytoplasmic ribonuclease XRN4 [49, 67–70]. To assess consistency with previous studies, we compare all 5′ features identified here with nanoPARE datasets generated from floral buds of wild type and *xrn4* mutants [51]. This analysis revealed an 85.9% overlap (*P* < 2.2e-16 using fisher’s exact test) in detected 5′ features (Supplementary Fig. S3C), confirming reproducibility of this approach.

### Capped 5′ features are concentrated around transcription start sites

We next examined the genomic distribution of 5′ features. The majority of 5′ features were associated with protein-coding genes [10 456 (92.9%) in wild type; 13 082 (92.6%) in *dcp2*], aligning predominantly within annotated promoters or 5′ UTRs (Fig. 2C). The presence of a 5′ cap typically marks a TSS, which is generally located within a transcription start region encompassing several alternative TSSs. Alternative TSS usage contributes to transcript isoform diversity, representing an important mechanism influencing post-transcriptional regulation and protein function [71, 72]. In this analysis, each 5′ feature can be considered equivalent to a transcription start region, while the 5′ cap position is the peak of uuG within a feature that correspond to a TSS. We assessed the positional distribution of 5′ caps in protein-coding genes by comparing their locations with transcript annotations from AtRTD3. AtRTD3 is a curated *Arabidopsis* reference transcriptome assembled from multiple experimentally derived transcript and TSS datasets [54]. The majority of 5′ caps were located at or near annotated TSSs (Fig. 2D). Examination of individual genes confirmed the 5′ caps were typically positioned at the annotated start site (e.g. *RPL35*) or represented alternative capped positions within gene bodies (e.g. *SNF1*-related protein kinase) (Fig. 2E). Further comparison revealed that 5′ features were slightly broader in *dcp2* compared with the wild type, with a median width of 124 and 103 nt, respectively (Supplementary Fig. S3D). Broader 5′ features in *dcp2* may indicate a greater number of TSSs distributed across a wider span. This suggests that in wild type, decapping followed by rapid degradation may restrict the accumulation of a subset of transcripts originating from such alternative TSSs.

### Genome-wide landscape of 5′-end caps in *dcp2* mutants

**Figure 3.**
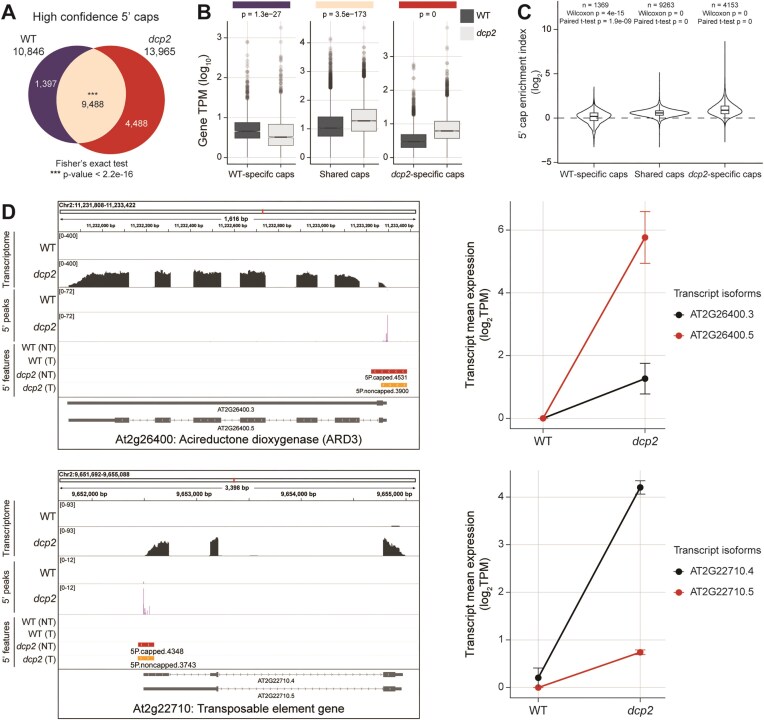
Genome-wide landscape of capped 5′-ends in *dcp2* mutants. (**A**) Venn diagram showing the comparison between high-confidence 5′ caps identified in wild type and *dcp2*. The overlapping region (beige) represents the caps shared between the samples, while the non-overlapping regions depict 5′ caps unique to either wild type (purple) or *dcp2* (red). (**B**) Boxplot showing the expression (log_10_TPM) of genes that are wild-type-specific capped genes (1397; in purple), shared capped genes (9488; in beige), or *dcp2*-specific capped genes (4488; in red).(**C**) Violin plot showing the distribution of 5′ cap enrichment index (log_2_) for capped genes classified as wild-type-specific, shared, or dcp2-specific. Statistical significance was assessed using paired Wilcoxon rank-sum test and paired *t*-tests, with *P*-values shown. (**D**) IGV browser track showing the coverage (y-axis) of transcriptome and 5′ peaks of *dcp2*-specific genes with example of ARD3 (At2g26400) and a transposable element gene (At2g22710) along with the expression of transcript isoforms (on right). The 5'-ends as identified in each sample and the gene structure of encoded transcripts [UTRs (narrow rectangle), exons (broad rectangle), introns (lines connecting the exons), and arrows indicate the orientation of the gene] from IGV are displayed. IGV, Integrated genome viewer; NT, No treatment; T, DCP2-treatment; TPM, Transcripts per million; WT, wild type.

To focus the analysis on biologically relevant capped 5′-ends, we restricted the downstream analyses to the untreated samples and used the treated libraries solely as a quality-control step. We therefore defined high-confidence capped TSSs as untreated capped features that lost their cap following *in vitro* DCP2 treatment, yielding 10 846 wild-type and 13 965 *dcp2* high-confidence capped features (Supplementary Tables S8 and S9). While 9488 capped sites (*P* < 2.2e-16 using fisher’s exact test) are shared, *dcp2* accumulates over 4400 unique capped genes than wild type (1397) (Fig. 3A). The large, shared cap fraction likely reflects a stable core TSS landscape and strong assay concordance. The quantitative analysis of gene expression further shows that *dcp2*-specific capped genes exhibit significantly higher steady-state levels compared to wild type (Fig. 3B, *P* = 0). Next, we calculated 5′ cap enrichment indices to compare the abundance of 5′-end signal over full-length gene in *dcp2* relative to wild type. We used the 5′ cap enrichment index as a proxy to quantify changes in cap signal in absence of DCP2 as described in “Materials and methods” section. The strongest 5′ cap enrichment index was observed for *dcp2*-specific capped genes (*n* = 4153; Fig. 3C), confirming that these genes are heavily capped (or stabilized in their capped state) when the decapping machinery is compromised. Shared capped genes (*n* = 9263) also displayed a positive enrichment index (Fig. 3C), suggesting that even capped genes detected in both genotypes exhibit a stronger cap signal in *dcp2* compared with wild type. In contrast, wild-type-specific caps (*n* = 1369) showed the weakest 5′ cap enrichment, implying no or small increases in cap accumulation in *dcp2*.

Further, we confirmed that transcripts with *dcp2*-specific caps showed low or undetectable expression in wild type, as indicated by global expression analysis (Fig. 3B) and exemplified by the cap footprints and transcript abundance of selected genes (Fig. 3D and Supplementary Fig. S4A).

Collectively, these examples suggest that DCP2-dependent decapping influences not only bulk RNA decay but also transcript isoform composition and accumulation of non-canonical capped genes. The data also indicate that many of these genes are normally eliminated rapidly through DCP2-mediated decay before they accumulate to detectable levels. Thus, these capped RNAs were not limited to altered abundance of annotated transcripts. Instead, the dataset uncovered a previously hidden layer of capped transcriptional output that becomes visible only when decapping is impaired.

Consistent with this idea, an analysis of published RNA decay data showed that *dcp2*-specific capped genes display increased stability and reduced decay rates in another decapping-deficient mutant *vcs-7* (Supplementary Fig. S4B) [10]. Notably, we identified 275 *dcp2*-specific capped loci within the intergenic regions (Supplementary Fig. S5 and Supplementary Table S9). These loci do not overlap with currently annotated genes and likely represent previously unannotated short transcripts that are normally highly unstable under wild type conditions.

Furthermore, inspection of 5′ caps localized near annotated TSSs revealed a marked increase in genes containing two or more alternative 5′ capped features in the *dcp2* mutant compared with the wild type (379 versus 150; Supplementary Fig. S6). Transcript-level analysis together with inspection of cap footprints indicated that these distinct 5′-end profiles frequently corresponded to the accumulation of different transcript isoforms between the two genotypes (Supplementary Fig. S6). These observations suggest that a subset of alternatively capped transcript isoforms is normally unstable and maintained at low abundance through DCP2-dependent decapping.

Conversely, wild-type-specific caps (1397) showed undetectable expression in *dcp2*, and notably, this set included 97 wild-type-specific capped loci located within intergenic regions. Representative examples are illustrated by the cap footprint profiles of selected genes and loci (Supplementary Fig. S7 and Supplementary Table S8).

### Cytosolic decay substrates are processed via canonical decapping

**Figure 4.**
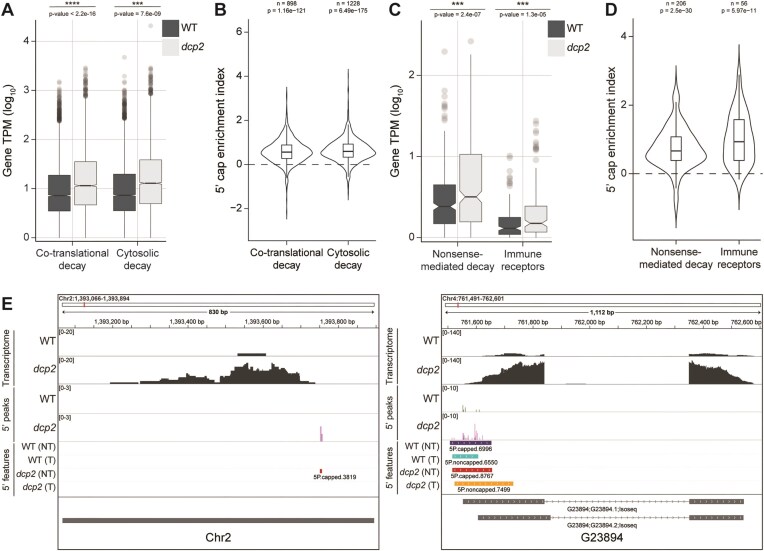
Cytosolic decay substrates are processed via canonical decapping. (**A**) Boxplot showing average gene expression (log_10_TPM) of co-translational and cytosolic decay targets that are expressed in our dataset. (**B**) Violin plot showing the distribution of 5′ cap enrichment index (log_2_) for genes undergoing co-translational or cytosolic decay target groups. Statistical significance was assessed using paired *t*-tests, with *P*-values shown. (**C**) Boxplot showing average gene expression (log_10_TPM) of NMD and immune receptor targets that are expressed in our dataset. (**D**) Violin plot showing the distribution of 5′ cap enrichment index (log_2_) for genes undergoing NMD decay. Statistical significance was assessed using paired *t*-tests, with *P*-values shown. (**E**) IGV browser track showing the coverage (*y*-axis) of transcriptome and 5′ peaks of selected NMD targets with example of a previously unannotated loci (left) and previously unannotated gene (G23894; on right). The 5′ features identified in each sample and the gene structure of encoded transcripts [UTRs (narrow rectangle), exons (broad rectangle), introns (lines connecting the exons), and arrows indicate the orientation of the gene] from IGV are displayed. *n*, number of genes analyzed per group; IGV, Integrated genome viewer; NT, No treatment; T, DCP2-treatment; TPM, Transcripts per million; WT, wild type.

Canonical decapping is proposed to function upstream of multiple cytoplasmic RNA decay pathways, including co-translational decay, cytosolic 5′–3′ turnover, and NMD [11–13, 30, 48, 67, 68, 73–76]. To directly test this relationship, we compared our capped dataset obtained in *dcp2* mutants with published degradome profiles describing XRN4-dependent co-translational and cytosolic decay substrates [68]. Among 12 306 genes detected in the degradome dataset, 11 441 were expressed in our datasets [transcript per million (TPM) ≥ 1]. Both co-translational and cytosolic decay targets showed elevated expression in *dcp2* compared with wild type (Fig. 4A). Many genes from both groups were also capped in both wild type and *dcp2* but showed higher 5′ cap enrichment in *dcp2* compared with wild type (Fig. 4B). Since XRN4 acts on 5′-monophosphorylated RNA, accumulation of capped transcripts in *dcp2* indicate that these RNA do not enter 5′–3′ decay when decapping is impaired. These findings positions DCP2 upstream of XRN4 within 5′–3′ turnover pathways.

Decapping-null seedlings phenotypically resemble NMD-deficient (*upf1*) mutants (Supplementary Fig. S1A) [76, 77]. Also, transcriptome profiling of *dcp2* revealed induction of defense and stress responses (Fig. 1C), a hallmark of NMD deficiency, which prompted further investigation. In plants, Enhanced Disease Susceptibility 1 (EDS1) acts as a central signaling hub during pathogen attack and coordinates other immune components and pathways to ensure a balanced defense response [78–80]. Because suppressing immune signaling rescues the growth defects of NMD mutants [76], we tested whether reducing EDS1-dependent immune signaling alleviates decapping-related growth defects. However, *vcs6 eds1* double mutants showed severe developmental defects similar to decapping single mutants, indicating that seedling lethality associated with impaired decapping cannot be explained solely by elevated immune responses (Supplementary Fig. S8).

To further examine the relationship between NMD and decapping, we analyzed a previously defined set of 333 high-confidence NMD targets together with 172 immune receptor genes enriched among NMD-regulated transcripts [76]. Most of these NMD-regulated genes were detected in our dataset and accumulated to higher levels in *dcp2* mutants compared with wild type (Fig. 4C). Importantly, these decay target groups also showed significantly elevated 5′ cap enrichment in *dcp2* compared with wild type (Fig. 4D), suggesting substrate overlap between NMD and decapping pathways. Beyond annotated NMD substrates, we additionally identified previously uncharacterized transcript isoforms and intergenic capped loci that accumulated specifically in both *upf1* and *dcp2* mutants (Fig. 4E). These observations support the conclusion that, in *dcp2*, rapidly degrading NMD targets are stabilized due to impaired decapping (Fig. 4C and D), leading to their accumulation. However, while decapping and NMD coregulate an overlapping subset of transcripts, *dcp2* seedling lethality does not appear to stem exclusively from misexpression of NMD-regulated immune receptors, but rather from a broader disruption of mRNA stability.

## Discussion

The 5′ cap plays a central role in determining the stability and fate of an RNA molecule [81]. In plants, bulk of cytoplasmic RNA decay is mediated by DCP2-dependent decapping followed by a 5′–3′ exonucleolytic decay pathway [10, 68]. This study provides a genome-scale, nucleotide-resolved view of DCP2-dependent 5′ caps in *A. thaliana* seedlings and positions canonical decapping at the center of multiple cytoplasmic RNA turnover pathways. By combining an *in vitro* decapping with nanoPARE-based 5′-end profiling, we resolved capped RNA 5′-ends at nucleotide resolution. While the global distribution of capped features near annotated TSSs is consistent with previous studies, our analysis extends beyond steady-state TSS mapping by identifying capped RNAs whose accumulation is sensitive to the loss of decapping activity.

A major finding of this study is that disruption of DCP2 leads to a marked increase in the diversity and abundance of detectable capped 5′-ends. In addition to canonical capped genes, *dcp2* mutants accumulated capped RNAs derived from low-abundance genes, alternative transcript isoforms, and previously unannotated intergenic loci. Many of these capped RNAs were undetectable or present at minimal levels in wild type, indicating that they are normally short-lived and efficiently removed through decapping-dependent turnover. Thus, rather than simply affecting bulk mRNA degradation, canonical decapping continuously restricts the accumulation of a broader and previously hidden repertoire of capped RNAs.

Importantly, our analysis reveals a differential distribution of 5′ caps between wild type and *dcp2*, which corresponded to differential accumulation of transcript isoforms as well as alternate TSSs between the genotypes. Alternative TSS usage is particularly important for modulating post-transcriptional regulation [71, 72]. Changes in transcript abundance may reflect a combination of direct stabilization of rapidly degraded mRNAs and secondary transcriptional consequences associated with impaired decapping and the severe developmental phenotype of *dcp2* mutants. Although the present analysis does not fully disentangle primary decapping-sensitive transcripts from the downstream indirect effects on gene expression, the increased cap enrichment indices are consistent with stabilization of capped transcripts resulting from impaired decapping activity. Indeed, *dcp2* mutants showed a prominent increase in 5′ caps and associated transcripts, including rare mRNAs and transcripts originating from previously unannotated loci. Their enrichment in *dcp2* mutants demonstrates that canonical decapping continuously suppresses a broader repertoire of capped RNAs than previously known. Moreover, many of these capped RNAs are barely detected in wild type, and their stabilization in *dcp2* therefore suggests that a subset of alternatively capped transcript isoforms is normally subject to rapid DCP2-dependent turnover and therefore persists only at very low steady-state levels.

In the cytoplasm, once decapped, transcripts become substrates for 5′–3′ exonucleolytic degradation by the XRN4 enzyme, thereby strongly influencing overall mRNA turnover [49, 67–70, 73]. The accumulation of capped genes from previously defined XRN4 substrates further positions DCP2 upstream of multiple cytoplasmic decay pathways. Both co-translational- and cytosolic-decaying XRN4-dependent genes showed increased cap enrichment in *dcp2*, consistent with failure to generate the 5′-monophosphorylated ends required for exonucleolytic degradation [48, 67, 68]. These findings provide an *in vivo* support for a model in which decapping is a prerequisite for both co-translational and cytosolic 5′–3′ decay pathways.

In addition, our data revealed a partial overlap between canonical decapping and NMD. A substantial fraction of high-confidence NMD targets, including immune receptor genes and previously unannotated unstable RNAs, accumulated as capped in *dcp2*. These observations support earlier models proposing that NMD substrates are frequently decayed through DCP2-dependent decapping [11, 74, 75]. However, genetic suppression of EDS1-dependent immune signaling did not rescue the developmental defects of decapping mutants, suggesting that the severe phenotypes associated with impaired decapping cannot be attributed solely to deregulation of NMD-associated immune targets. Instead, our findings indicate that loss of decapping disrupts multiple RNA surveillance and turnover pathways simultaneously, leading to widespread perturbation of transcriptome homeostasis. Our dataset should primarily be interpreted as a profile of decapping-sensitive capped RNAs rather than a complete representation of all decay intermediates.

In summary, this study provides a resource on 5′ cap distribution in *Arabidopsis* and establishes DCP2 as a central determinant of cap-defined RNA fate *in vivo*. Beyond its established role in bulk mRNA turnover, canonical decapping restricts the accumulation of unstable, cryptic, and non-canonical capped transcripts and interfaces with co-translational, cytosolic, and NMD-associated decay pathways. The cap footprints defined here provide a resource for future studies of isoform-specific RNA turnover, cryptic transcription, and the integration of decapping with parallel RNA surveillance pathways during development and stress responses.

## Supplementary Material

lqag088_Supplemental_Files

## Data Availability

All the sequencing data generated in this study have been deposited in the Gene Expression Omnibus (GEO) under accession number GSE315704.
